# Impact of data layouts on the efficiency of GPU-accelerated IDW interpolation

**DOI:** 10.1186/s40064-016-1731-6

**Published:** 2016-02-01

**Authors:** Gang Mei, Hong Tian

**Affiliations:** School of Engineering and Technolgy, China University of Geosciences, No. 29 Xueyuan Road, Beijing, 100083 China; Institute of Earth and Environmental Science, University of Freiburg, Albertstr.23B, 79104 Freiburg im Breisgau, Germany; Faculty of Engineering, China University of Geosciences, No. 388 Lumo Road, Wuhan, 430074 China

**Keywords:** GPU, Data layout, IDW interpolation, CUDA dynamic parallelism

## Abstract

This paper focuses on evaluating the impact of different data layouts on the computational efficiency of GPU-accelerated Inverse Distance Weighting (IDW) interpolation algorithm. First we redesign and improve our previous GPU implementation that was performed by exploiting the feature of CUDA dynamic parallelism (CDP). Then we implement three versions of GPU implementations, i.e., the naive version, the tiled version, and the improved CDP version, based upon five data layouts, including the *Structure of Arrays* (SoA), the *Array of Structures* (AoS), the *Array of aligned Structures* (AoaS), the *Structure of Arrays of aligned Structures* (SoAoS), and the *Hybrid* layout. We also carry out several groups of experimental tests to evaluate the impact. Experimental results show that: the layouts AoS and AoaS achieve better performance than the layout SoA for both the naive version and tiled version, while the layout SoA is the best choice for the improved CDP version. We also observe that: for the two combined data layouts (the SoAoS and the Hybrid), there are no notable performance gains when compared to other three basic layouts. We recommend that: in practical applications, the layout AoaS is the best choice since the tiled version is the fastest one among three versions. The source code of all implementations are publicly available.

**Electronic supplementary material:**

The online version of this article (doi:10.1186/s40064-016-1731-6) contains supplementary material, which is available to authorized users.

## Introduction

Data layout is the form in which data should be organized and accessed in memory when operating on multi-valued data such as sets of 3D points. The selecting of appropriate data layout is a crucial issue in the development of graphics processing unit (GPU) accelerated applications. The efficiency performance of the same GPU application may drastically differs due to the use of different types of data layout; see the example of sorting structures demonstrated with *Thrust* (Bell and Hoberock 2012).

**Fig. 1:**
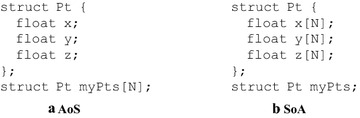
Data layouts: Array-of-Structures (AoS) and Structure-of-Arrays (SoA) **a** AoS; **b** SoA

Typically, there are two major choices of the data layout: the *Array of Structures* (AoS) and the *Structure of Arrays* (SoA) (Farber 2011); see Fig. 1. Organizing data in AoS layout leads to coalescing issues as the data are interleaved. In contrast, the organizing of data according to the SoA layout can generally make full use of the memory bandwidth due to no data interleaving. In addition, global memory accesses based upon the SoA layout are always coalesced. The above two layouts are probably the most basic and simplest memory access patterns. More complex data layouts such as the *Array of Structures of Arrays* (AoSoA) (Abel et al. 1999) and the *Structure of Arrays of Structures* (SoAoS) (Siegel et al. 2009) can be formed by combining the basic layouts AoS and SoA.

As noted above, the memory access patterns are critical for the performance of GPU-accelerated applications. However, it is not always obvious which data layout will achieve better performance for a specific GPU application. For example, to evaluate the performance of the SoA and AoS layouts, Govender et al. (2014) ran a simulation of 2 million particles using their discrete element simulation framework, and found that AoS is three times slower than SoA, while an opposite argument was presented in Giles et al. (2013). In the library framework OP2, Giles et al. (2013) preferred to use the AoS layout to store mesh data for better memory accesses performance. In practice, a common solution is to implement a specific application using above two layouts separately and then compare the performance.

The inverse distance weighting (IDW) interpolation algorithm, which was originally proposed by Shepard (1968), is one of the most commonly used spatial interpolation methods in Geosciences. Typically, the implementation of spatial interpolation within the conventional sequential programming patterns is computationally expensive for a large number of data sets. In order to improve the computational efficiency, some efforts have been carried out to develop efficient implementations of the IDW interpolation in various massively parallel computing environments on multi-core CPUs (Armstrong and Marciano 1997; Guan and Wu 2010; Huang et al. 2011) and/or GPUs platforms (Hanzer 2012; Hennebőhl et al. 2011; Huraj et al. 2010; Xia et al. 2011).

In our previous work (Mei 2014), we presented two GPU implementations of the standard IDW interpolation algorithm with the compute unified device architecture (CUDA), including the tiled version that took advantage of shared memory and the CDP version that was implemented by exploiting CUDA dynamic parallelism (CDP). We found that the tiled version achieved the highest speedups over the CPU version. However, the naive GPU version is 4.8–6.0 times faster than the CDP version. Those experimental tests were performed only on single precision.

In this paper, we focus on evaluating the performance impact of different data layouts when implementing the IDW interpolation on the GPU. We first redesign the CDP version to avoid the use of the atomic operation atomicAdd(), and then test three GPU implementations, i.e., the naive GPU version presented in Huraj et al. (2010), the tiled version described in Mei (2014), and the improved CDP version introduced in this paper, on single precision and/or double precision. In our previous work (Mei 2014), the above three GPU implementations are developed according to the data layout SoA. In order to evaluate the impact of other data layouts such as AoS, we also implement these GPU versions based upon the AoS layout and other combined data layouts such as SoAoS (Siegel et al. 2009), and then test their performance on single and/or double precision.

In summary, we make the following contributions in this paper:

1. Redesigning the CDP version that is originally presented in Mei (2014), to improve its efficiency;
2. Implementing five groups of those three GPU versions based upon five different data layouts on both single and/or double precision;
3. Evaluating the impact of five data layouts on the computational efficiency of three versions of GPU-accelerated IDW interpolation on single and/or double precision.

This paper is organized as follows. “Background” section gives a brief introduction to the IDW interpolation and two basic data layouts, the SoA and AoS. “GPU implementations” section concentrates on the GPU implementations that are performed by using five different data layouts. “Results and discussion” section presents some experimental tests that are performed on single and/or double precision, and discusses the experimental results. Finally, “Conclusion” section draws some conclusions.

## Background

In this work, the major research objective is to evaluate the impact of data layouts on the efficiency of GPU-accelerated IDW interpolation. Before describing our GPU-accelerated implementations of the IDW algorithm, in this section we first briefly introduce the principal of the underlying algorithm, IDW interpolation, and commonly used data layouts.

### IDW interpolation

The IDW algorithm is one of the most commonly used spatial interpolation methods in Geosciences, which calculates the interpolated values of unknown points (prediction points) by weighting average of the values of known points (data points). The name given to this type of methods was motivated by the weighted average applied since it resorts to the inverse of the distance to each known point when calculating the weights. The difference between different forms of IDW interpolation is that they calculate the weights variously.

A general form of predicting an interpolated value *Z* at a given point *x* based on samples $Z_{i}=Z(x_{i})$ for *i* = 1, 2, ..., *n* using IDW is an interpolating function:

1 $$\begin{aligned} Z(x)=\sum \limits _{i=1}^n {\frac{\omega _i (x)z_i }{\sum \nolimits _{j=1}^n {\omega _j (x)} }} , \quad \omega _i (x)=\frac{1}{d(x,x_i )^p} \end{aligned}$$

The above equation is a simple IDW weighting function, as defined by Shepard (1968), where *x* denotes a predication location, $x_{i }$ is a data point, *d* is the distance from the known data point $x_{i}$ to the unknown prediction point *x*, *n* is the total number of data points used in interpolating, and *p* is an arbitrary positive real number called the power parameter (typically, $p = 2$).

### Data layout

In GPU computing, an optimal pattern of accessing data can significantly improve the overall efficiency performance by minimizing the number of memory transactions on the off-chip global memory. Thus, one of the key design issues for generating efficient GPU code is the selecting of proper data layouts when operating on multi-valued data such as sets of points or pixels. In general, there are two major choices of the data layout: the AoS and the SoA; see Fig. 1.

Organizing data in AoS layout leads to coalescing issues as the data are interleaved. Multi-dimensional and multi-valued data containers lead to strided memory accesses in the one dimensional address space, and cause exactly this problem. For example, performing an operation on a set of 3D points illustrated in Fig. 1a that only requires the variable *x* will result in about a 66 % loss of bandwidth and waste of L2 cache memory.

In contrast, the organizing of data according to the SoA layout can typically make full use of the memory bandwidth since there is no data interleaving; see Fig. 1b. Furthermore, global memory accesses are always coalesced when using this type of data layout; and usually higher global memory performance can be achieved.

The SoA data layout is beneficial in many cases. Farber (2011) suggested that from a GPU performance perspective, it is preferable to use the SoA layout. This argument was demonstrated by the example of sorting SoA and AoS structures with *Thrust*; it was reported that a five-times speedup can be achieved by using a SoA data structure over a AoS data structure (Bell and Hoberock 2012).

Similarly, in order to gauge the effective performance of the two representations, i.e., the SoA and AoS layouts, on the GPU, Govender et al. (2014) ran a simulation of 2 million particles using their discrete element simulation framework BLAZE-DEM, and found that AoS is three times slower than SoA.

However, an opposite argument was presented in Giles et al. (2013). In the library framework for the solution of unstructured mesh applications OP2, Giles et al. (2013) and Mudalige et al. (2013) preferred to use the AoS layout to store mesh data for better memory accesses performance.

The above mentioned applications indicate that memory access patterns (e.g., AoS and SoA) are critical for performance, especially on parallel architectures such as GPUs. However, it is not always obvious which data layout will achieve better performance in a particular application. The selection of a proper data layout for a specific application depends on its underlying algorithm. In general, the usual language syntax and standard container types lead naturally to the AoS layout while SIMD units much prefer the SoA format (Strzodka 2012a).

In order to improve the efficiency of accessing memories on the GPU, many studies have been performed to transform different types of layouts to others, e.g., from AoS to SoA, or vice versa (Mistry et al. 2011; Strzodka 2012a, b; Sung et al. 2012). Furthermore, the major choices of AoS and SoA can be further refined to form hybrid formats such as AoSoA (Abel et al. 1999) and SoAoS (Siegel et al. 2009).

In this work, we will evaluate the performance impact of the above two basic data layouts and other layouts that are derived from the above two layouts. A group of GPU implementations of those three versions will be developed particularly by using one type of data layout, and then compared to other groups of implementations.

## GPU implementations

In this section, we will first present three versions of the GPU-accelerated IDW implementation, i.e., the naive version presented in Huraj et al. (2010), the tiled version described in Mei (2014), and the improved CDP version introduced in this paper, and then describe five groups of the GPU implementation that are developed by the use of five types of data layout.

### Three versions of the GPU implementation

#### The naive version

The naive version is a straightforward implementation of the IDW interpolation, in which only registers and global memory are used without taking advantage of shared memory (Armstrong and Marciano 1997). More specifically, when *m* data points are accepted to calculate the interpolated values for *n* prediction points, *n* threads are needed to be allocated; and each thread takes the responsibilities for calculating the distances from one prediction point to all of those *m* data points, the corresponding inverse weights, and the weighted average. Note that in this version each thread needs to load the coordinates of *m* data point from global memory. Therefore, the coordinates of all data points are needed to be read *n* times.

#### The tiled version

The tiled version is implemented by taking advantages of the shared memory with the use of the optimization strategy “tiling” (NVIDIA 2015). In this version, the coordinates of data points is first transferred from global memory to shared memory; then each thread within a thread block can access the coordinates stored in shared memory concurrently.

More specifically, each thread invoked to first load the coordinates of one data point from global memory to shared memory, and then compute the distances and inverse weights to those data points stored in current shared memory. After the completion of calculating these partial distances and weights, next “tile” of data residing in global memory is newly loaded to shared memory and then accepted to compute current round of partial distances and weights. Each thread accumulates the result of all partial weights and all weighted values into two registers. Finally, the desired interpolation value of each prediction point can be obtained according to the sums of all partial weights and weighted values.

By adopting the optimization strategy “tiling”, the global memory accesses can be significantly reduced for that the coordinates of data points are only needed to be read (*n* / threadsPerBlock) times rather than *n* times from global memory, where threadsPerBlock is the number of threads within a thread block.

#### The CDP version

The CDP version of the GPU implementation is the one that is implemented by adopting the feature CDP. In our previous work (Mei 2014), we have developed the CDP implementation of the standard IDW interpolations. In this section, we will describe an improved version of the CDP implementation.

The CDP version presented in Mei (2014), which is referred to as the original CDP version in this section, has two levels of nested parallelism: (1) level 1: for all prediction points, the interpolated values can be calculated in parallel; (2) lever 2: for each prediction point, the distances to all data points can be calculated in parallel. The parent kernel is responsible to performing the first level of parallelism, while the child kernel takes responsibility for realizing the second level of parallelism.

In the original CDP version, each child kernel is responsible to calculating the distances from all data points to a predication point. More specifically, first each thread within a child grid is invoked to calculate: (1) the distance from one data point to a predication point, (2) the corresponding weight, and (3) the weighted value [see Eq. (1)]; and then the weights and weighted values calculated within the same thread block will be locally accumulated using the parallel reduction (Harris 2007); finally all weights and weighted values that have been obtained within different blocks will be accumulated using the atomic operation atomicAdd().

The atomic operations such as atomicAdd() cannot be performed on double precision. In order to enable the CDP version to be executed on double precision, we redesign and improve this GPU implementation to avoid the use of atomicAdd(). The basic idea behind this improvement is as follows.

In the improved CDP version, we no longer allocate *n* threads within a child grid (where *n* is the number of data points), but only allocate one thread block with 1024 threads. Within this single thread block, each thread is responsible to calculating the distances of several data points rather than only one data point to a predication point. For example, assuming there are 3000 data points, for each predication point, it is needed to calculate all the distances from the predication point to those 3000 data points. Each thread will take responsibilities for calculating three [i.e., $(3000 + 1024 - 1) / 1024 = 3$] distances. These three distances and corresponding weights will be locally accumulated within each thread; and when all threads within the only one block finish calculating all distances, the accumulation of all weights and weighted values will be achieved by performing a parallel reduction (Harris 2007) within the thread block. Thus, in this solution, the operation atomicAdd() is not needed for accumulating all weights and weighted values that have been calculated within different blocks of threads.

We test the performance of the improved CDP version using five sets of data. In each set of test data, the numbers of data points and predication points are to be identical. We create five groups of sizes, i.e., 10, 50, 100, 500, and 1000 K (1 K = 1024). And five tests are performed by setting the numbers of both the data points and prediction points as the above listed five groups of sizes.

**Fig. 2:**
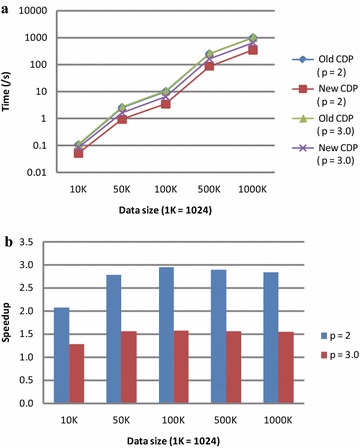
Performance comparison of the original (old) and the improved (new) CDP versions. **a** Execution time of the new and old versions; **b** speedups of the new version over the old version

The performance of the original and the improved CDP versions is illustrated in Fig. 2. These experimental tests show that the improved CDP version achieves the speedups of 2.9 and 1.5 over the original CDP version when the power parameter *p* is set to 2 and 3.0, respectively. Noticeably, for the original CDP version, the performance in the two cases where the power parameter *p* is set as 2 and 3.0 is almost the same; thus, in the Fig. 2a, the two lines representing the execution time of the old version are almost overlapped.

### Five groups of the GPU implementation based upon five data layouts

In this paper, the naive version presented in Huraj et al. (2010), the tiled version developed in Mei (2014), and the improved CDP version described above are accepted to be implemented according to five data layouts for benchmark tests on single precision and/or double precision.

#### The SoA group of implementations

We develop the naive version, the tiled version, and the improved CDP version of the GPU implementations according to the layout SoA. The coordinates of the data points and the prediction points are expected to be stored in two structures of arrays, respectively; see Fig. 1b. However, in practical implementations, due to the fact that there are only two structures of arrays and in fact six arrays (i.e., $2 \times 3 = 6$) needed, we directly use six arrays, e.g., dx[n], dy[n], dz[n], px[n], py[n], and pz[n], to store the coordinates of data points and prediction points.

In our previous work (Mei 2014), we have introduced three GPU implementations of the standard IDW interpolations, i.e., the naive version, the tiled version, and the original CDP version. These GPU implementations are completely developed according to the data layout SoA. We slightly modify the naive version and the tiled version of implementations, and additionally develop the improved CDP version according to the layout SoA.

#### The AoS group of implementations

The implementations of the three GPU versions according to the layout AoS is quite straightforward, which can be realized by simply modifying the SoA group of the three GPU implementations. First, two arrays of structures that are used to store the coordinates of all data points and predications points are allocated; and then the references to the points’ coordinates in the SoA version of the GPU implementations are replaced by using the arrays that are represented in the AoS format.

Note that, in this group of implementations, the structure for representing 3D points are misaligned; in other words, the structure is not forced to be aligned using the specifier __align__(); see Fig. 1a.

#### The AoaS group of implementations

In the AoS group of GPU implementations described above, the data structure for representing 3D points is not forced to be aligned. Operations using the misaligned structure may requires much more memory transactions when accessing global memory, and thus decreases the overall efficiency performance (NVIDIA 2015).

**Fig. 3:**
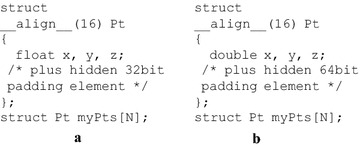
Data layout: Array of aligned Structures (AoaS). **a** Single precision; **b** double precision

In order to benefit from the aligned memory accesses, we simply add the specifier __align__ into the data structures; see Fig. 3. Noticeably, on single precision, a hidden 32 bit (i.e., $128 - 3 \times 32 = 32$) padding element is implicitly inserted into the structure Pt to meet the 128 bit size requirement for alignment; while on double precision, the hidden padding element is 64 bit, and the access to this structure needs two 128 bit read or write instructions (i.e., $64 \times 3 + 64 = 2 \times 128$).

**Fig. 4:**
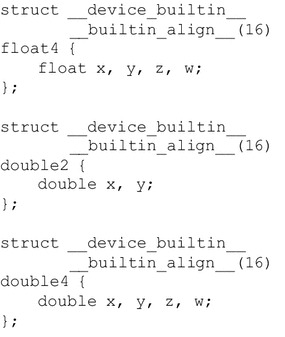
Several build-in data types in CUDA. (The data type double4 is aligned into two 16 bytes words)

Another notable issue in exploring the AoaS layout is the use of build-in data types. CUDA has provided various build-in data types; see Fig. 4 for three examples. The size requirement for alignment is automatically fulfilled for some built-in data types like float2, float4, or double2.

We also use the build-in types float4 and double4 to develop a *build-in* group of GPU implementations. This build-in group of GPU implementations is quite easily implemented by replacing the structure Pt with float4 or double4. The only difference between the AoaS format data types illustrated in Fig. 3 and those build-in types shown in Fig. 4 is that: in the AoaS format data types, a hidden padding element is implicitly added, while the component w is explicitly defined in float4 or double4 to be used as a padding element.

**Fig. 5:**
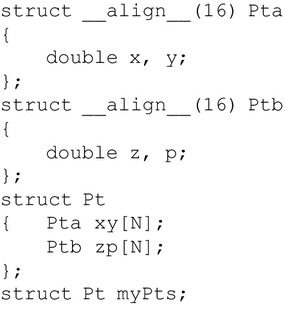
Data layout: Structure of Arrays of aligned Structures (SoAoS)

**Fig. 6:**
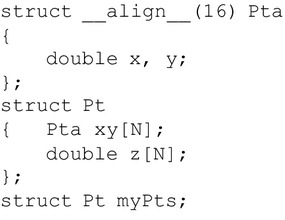
The hybrid data layout by combining AoS and AoV

We test the build-in group of GPU implementation and compare the performance with that of the AoaS group. We find that there are no remarkable performance gains. Hence, we do not adopt these build-in data types to form combined data types, but choose the user-defined data types to create hybrid types; see Figs. 5 and 6.

#### The SoAoS group of implementations

When operating on structures residing in global memory, typically there are two major optimization strategies (Siegel et al. 2009):

- Accessing consecutive elements to guarantee for coalesced reads.
- Alignment of data structures to allow for fewer reads.

The above two strategies can generally achieve performance improvements in the use of global memory. In order to benefit from both methods, a combined data layout, SoAoS, is proposed in Siegel et al. (2009). By organizing aligned structures that don’t exceed the alignment boundary in multiple arrays, it is able to reduce the overall number of issued reads by using 64 or 128 bit memory accesses while guaranteeing that all the memory accesses of the single threads within the same warp (or half-warp on some devices) are coalesced; see Fig. 5. Note that the component p in the structure Ptb is just an explicit padding element that will never be used in calculating.

The data structures illustrated in Fig. 5 are particularly designed for double precision. In this paper, we only implement the three GPU implementations of the IDW interpolation on double precision according to the SoAoS layout and related data structures. We do not implement the three GPU implementations on single precision according to the SoAoS layout. The reasons why we do not develop the implementations are as follows:

We attempt to test the simple IDW interpolation in this paper; and for this simple IDW interpolation, the input and output data are only the coordinates of 3D points without any needed additional information.

On single precision, the coordinates of a 3D points, i.e., float x, y, z, needs 12 byte (4 byte $\times$ 3 values); and in CUDA the memory size for alignment is maximum 16 byte. Thus, it only need one time of the memory size for alignment because 12 byte is less than the maximum memory size for alignment, i.e., 16 byte.

In other words, we can only use ONE aligned structure Pt to store the coordinates float x, y, z, see Fig. 3. In this case, the implementations are in fact developed according to the layout AoaS, while we have implemented the three GPU implementations according to the layout AoaS on single precision.

#### The hybrid group of implementations

The SoAoS layout described above is a combination of the layouts SoA and AoS. In this paper, specifically for the IDW interpolation, we also introduce another combined data layout which is a combination of the AoS and the *Array of Values *(AoV); see Fig. 6. A major difference between this hybrid layout and the SoAoS layout is the use of an AoV format array (i.e., double z[N] in Fig. 6) to replace an AoS format array (i.e, Ptb zp[N] in Fig. 5). Another difference is that in this hybrid layout there is no explicit or implicit padding element.

Similar to the layout SoAoS, the hybrid layout is only applicable on double precision. In the above “The SoAoS group of implementations” section, We have explained the reasons why not implement the three GPU implementations on single precision according to the combined layouts. Thus, we also implement the three GPU implementations only on double precision according to this hybrid layout and related data structures illustrated in Fig. 6.

## Results and discussion

### Results

The GPU implementations are evaluated using the NVIDIA GeForce GT640 (GDDR5) graphics card and the CUDA 5.5. Note that the GeForce GT640 card with memory GDDR5 has the Compute Capability 3.5, while it only has Compute Capability 2.1 with the memory DDR3. For each set of the testing data, we carry out all GPU implementations on single precision and/or double precision.

For the CPU implementations, we directly adopt our previous results that were performed on single precision. These results have been presented in Mei (2014); and in this paper, they are directly accepted to be used as the baseline. The efficiency performance of all GPU implementations is benchmarked by comparing to the baseline results.

As described in Mei (2014), for each GPU implementation, we tested two different forms that have different values of the power parameter *p*. In the first form, the power *p*, see Eq. 1, is set to an integer value 2, while this value is set to 3.0 in the second form. In this paper, we only consider the first form (i.e., $p = 2$).

The input of the IDW interpolation is the coordinates of data points and prediction points. The performance of the CPU and GPU implementations may differ due to different sizes of input data (Hanzer 2012; Hennebőhl et al. 2011). However, the motivation of this work is focused on evaluating the performance impact of different data layouts; thus, we only consider a special situation where the numbers of prediction points and data points are identical.

We create five groups of sizes, i.e., 10, 50, 100, 500, and 1000 K (1 K = 1024). And five tests are performed by setting the numbers of both the data points and prediction points as the above listed five groups of sizes.

#### Single precision

**Fig. 7:**
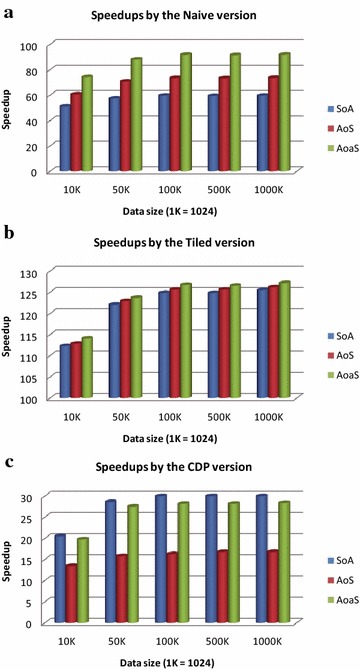
Performance of GPU implementations on single precision

On single precision, we implement those three GPU implementations of the IDW interpolation using three types of data layouts, including the SoA, the AoS, and the AoaS. The benchmark results (i.e., speedups generated by comparing to the baseline CPU results) of the naive version, the tiled version, and the CDP version are shown in Fig. 7.

According to the results generated in above three experimental tests, we have found that: for both the naive and tiled implementations, the layout AoaS achieves the best performance and the layout SoA obtains the worst results; see Figs. 7a, b. However, for the CDP version, the layout SoA achieves the best performance, and the second best is the layout AoaS, while the AoS layout leads the worst results; see Fig. 7c.

#### Double precision

**Fig. 8:**
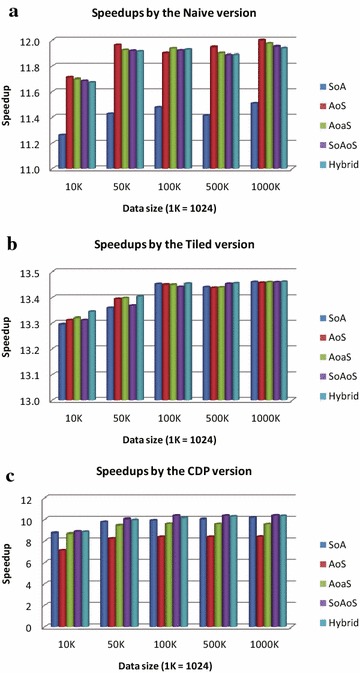
Performance of GPU implementations on double precision

On double precision, we implement two groups of those three GPU implementations using additional two types of combined data layouts, the SoAoS and the Hybrid; we also implement the GPU implementations using the layouts SoA, AoS, and AoaS. The experimental results in this case are presented in Fig. 8.

For the naive version, the speedups generated by the GPU implementation according to the layout SoA are the lowest, while the other four layouts achieve almost the same performance although the speedups are slightly varied; see Fig. 8a.

For the tiled version, all of the five different data layouts obtain nearly the same performance. There are only several slight differences among those speedups; see Fig. 8b.

For the CDP version, the layout AoS leads the worst performance; and the second worst results are generated by the layout AoaS. The other three layouts including the SoA, the SoAoS, and the Hybrid obtain almost the same performance; see Fig. 8c.

### Discussion

Recently, the GPU-computing programming models such as CUDA are popularly used to speed up various scientific applications. However, fully utilizing the specific features of the underlying GPU architecture is still a challenging work. One of the most important responsibilities of a programmer is to maximize the efficiency performance by optimizing the memory hierarchy in GPU-computing.

The data layout in memory is a critical issue in developing efficient GPU code. Several efforts have been carried out to analyze (Giles et al. 2013; Siegel et al. 2009; Strzodka 2012a) or transform (Mistry et al. 2011; Strzodka 2012b; Sung et al. 2012) different types of data layouts.

Based upon our previous work (Mei 2014), in this paper we focus on evaluating the performance impact of different data layouts on the IDW interpolation. First, we develop five groups of the GPU implementations of the standard IDW interpolation according to five types of data layout, and then test their efficiency performance on single precision and/or double precision.

#### Impact of data layouts on single precision

On single precision, we implement three groups of the GPU implementations using three types of data layouts, including the SoA, the AoS, and the AoaS. We find that: for the AoS and AoaS layouts, the second one always obtains better performance than the first.

More specifically, the AoaS can achieve the average speedups of about 92, 126, and 28 for the naive, the tiled, and the CDP versions, respectively, while the AoS achieves the average speedups of about 73,124, and 16 for those three versions. The result also indicates that: the AoaS is about 1.25, 1.01, and 1.72 times faster than the AoS for those three versions. This positive impact on performance is due to minimizing the number of memory transactions by aligning the data structures.

We also observe that: the AoaS is about 1.25 and 1.72 times faster than the AoS for the naive version and the CDP version, respectively, but it is only 1.01 times faster than the AoS for the tiled version. This result also means that: for all of the three versions of GPU implementations, the performance impact due to the use of the AoaS over the AoS for both the naive version and the CDP version is much more significant than that for the tiled version.

The above performance result is perhaps because of the effective optimization in the use of global memory by minimizing the number of memory transactions. In both the naive version and the CDP version, each thread needs to read the coordinates of all data points; in other words, the coordinates of all data points are needed to be read *n* times, where *n* is the number of predication points. In contrast, the coordinates of all data points are only needed to be read (*n*/threadsPerBlock) times due to accepting the optimization strategy “*tiling*”. Thus, there are much more global memory accesses in both the naive and the CDP versions than that in the tiled version. And the impact of optimizing the use of global memory by minimizing the number of transactions on a larger number of global memory accesses is obviously more significant than that on a smaller number of global memory accesses.

The above two layouts (AoS and AoaS) achieve higher speedups than the layout SoA for both the naive and tiled implementations, but get lower speedups than the SoA for the CDP implementation. More specifically, the AoS and AoaS are 1.23 and 1.54 times faster than the SoA for the naive implementation, and 1.01 and 1.02 times faster than the SoA for the tiled version. However, in contrast, for the CDP implementation the SoA is about 1.09 and 1.81 times faster than the AoS and AoaS, respectively.

We cannot explain this strange behavior. Perhaps this behavior is due to the nested parallelism when programming with the feature of CDP.

Another notable issue in exploring the AoaS layout is the use of build-in data types. CUDA has provided various build-in data types. The size requirement for alignment is automatically fulfilled. Compared to those user-defined data types in AoaS formant (see Fig. 3), we have found the counterparts of the build-in data types provided by CUDA do not achieve notable advantages. In addition, the user-defined AoaS data types are suggested to be used for the convenience in programming.

#### Impact of data layouts on double precision

On double precision, we also observe some performance results that are as the same as those on single precision:

1. For the naive version, both the layouts AoS and AoaS are better than the SoA.
  As explained above, this positive result is because of aligning the data structures to allow for fewer reads or writes. However, the performance gains generated by using the layouts AoS and AoaS against the SoA on double precision are not as significant as those obtained on single precision. On single precision, the average speedups of the layouts SoA, AoS, and AoaS are about 59, 73, and 92, respectively, while on double precision, the average speedups are only about 11.42, 11.91, and 11.88.
2. For the tiled version, those three layouts, SoA, AoS, and AoaS achieve almost the same performance.
  This result is due to the fact that the accesses to global memory have been optimized using the strategy “tiling” and the impact of different data layouts on accessing global memory is not significant.
3. For the CDP version, the layout SoA still obtains best results when compared to the layouts AoS and AoaS.
  More specifically, the layout SoA is about 1.20 and 1.04 times faster than the layouts AoS and AoaS, respectively. We cannot give reasonable explanations for this strange behavior. We guess that the coalesced access to global memory in nested parallelism by exploiting the feature CDP has a very positive performance impact.

Furthermore, we find several additional results on double precision.

1. For the naive version, all the data layouts except the SoA achieve nearly the same speedups, i.e., about 11.88. Noticeably, among these four layouts, i.e., the AoS, the AoaS, the SoAoS, and the Hybrid, the best one is the AoS, in which the alignment is not used.
  This result is perhaps due to two reasons: the first is that the aligning for data structures on double precision is not as effective as that on single precision (see Figs. 7a, 8a); the second potential cause is that there is probably a performance penalty when aligning data structures on double precision. However, the advantage of the AoS layout is not obvious.
2. For all the three versions, the performance differences between the SoAoS layout and the Hybrid layout are quite small (<0.3 % of the average speedups). This illustrates that the use of the AoS or the AoV in a combined layout on double precision does not lead to heavy impact on performance.

##### *Remark*

On double precision, the influence of using different types of data layouts on computational efficiency is not as obvious as that on single precision. One of the potential causes is that: on double precision the performance gains in efficiency by exploiting GPU-acceleration are much less than those on single precision. In other words, the overall speedups obtained on double precision (i.e., about 8–14) are much lower than those achieved on single precision (i.e., about 15–130).

#### Recommendations and future work

Considering the overall efficiency performance on single and double precision, we recommend that: for both the naive version and the tiled version, the best choice is the data layout AoaS, while the layout SoA is the best one for the CDP version. From the perspective of GPU performance in practical applications, the layout AoaS is suggested to be the only option since that the tiled version is the fastest one among the three versions of GPU implementations. These recommendations are probably also valuable when accelerating other interpolation algorithms such as Kriging (Krige 1951) and DSI (Mallet 1992).

In this paper, all the experimental tests are performed and evaluated on a single GPU. In some related work (Guan and Wu 2010; Huang et al. 2011), efficient implementations of the IDW interpolation were developed on the platforms of multiple GPUs or on clusters. When intending to benefit from multi-GPUs or clusters, it is needed to carefully analyze and select the optimal data layout. Future work should therefore include the implementation of the IDW interpolation and the performance evaluation of different data layouts under the environment of multi-GPUs or clusters.

## Conclusion

We have redesigned and improved the CDP version of the GPU implementations of the standard IDW interpolation algorithm by exploiting the feature CDP in CUDA. We have demonstrated that the improved CDP version has the speedups of 2.9 and 1.5 over the original CDP version when the power parameter *p* is set to 2 and 3.0, respectively. In further, in order to evaluate the performance impact of different data layouts, we have implemented the naive version, the tiled version, and the improved CDP version based upon three basic layouts (SoA, AoS, and AoaS) and two combined layouts. We have observed that: (1) for both the naive version and tiled version, the layouts AoS and AoaS achieve better performance than the layout SoA; (2) for the improved CDP version, the layout SoA is the best choice among the three basic layouts; (3) for the two combined data layouts, there are no notable performance gains when compared to those three basic layouts. We recommend that: in practical applications, the layout AoaS is the best choice since the tiled version is the fastest one among the three versions of GPU implementations, especially on single precision. All the GPU implementations are publicly available (Additional files 1, 2, 3, 4, 5).

## Additional files

10.1186/s40064-016-1731-6 The SoA group of GPU implementations.
10.1186/s40064-016-1731-6 The AoS group of GPU implementations.
10.1186/s40064-016-1731-6 The AoaS Group of GPU implementations.
10.1186/s40064-016-1731-6 The SoAoS Group of GPU implementations.
10.1186/s40064-016-1731-6 The Hybrid Group of GPU implementations.

## Abbreviations

AoaS: Array of aligned Structures
AoS: Array of Structures
AoSoA: Array of Structures of Arrays
AoV: Array of Values
CDP: CUDA dynamic parallelism
CPU: central processing unit
CUDA: compute unified device architecture
GPU: graphics processing unit
IDW: inverse distance weighting
SoA: Structure of Arrays
SoAoS: Structure of Arrays of aligned Structures
